# Creating pelvic exenteration services: obstacles, cultural factors, and learning experiences from the first specialized service in Saudi Arabia and the Gulf region

**DOI:** 10.3389/fonc.2026.1781448

**Published:** 2026-03-12

**Authors:** Raha Alahmadi, Samar Alhomoud, Luai H. Ashari

**Affiliations:** 1 Section of Colorectal Surgery, Department of Surgical Oncology, King Faisal Specialist Hospital & Research Centre, Riyadh, Saudi Arabia; 2 College of Medicine, Alfaisal University, Riyadh, Saudi Arabia

**Keywords:** locally advanced rectal cancer (LARC), multi-disciplinary work, pelvic exenteration (PE), pelvic exenterative surgery, pelvic malignancies, recurrent rectal cancer

## Abstract

Pelvic Exenteration has evolved over the years into a multidisciplinary collaboration that requires structural elements and staffed roles beyond surgeons alone. Establishing such a service requires integrated multidisciplinary pathways, specialized imaging and anesthesia support, experienced perioperative nursing, stoma and wound care expertise, and administrative support, along with a culture that embraces centralization and high-risk oncologic surgery. Centers without previously existing exenteration programs face additional challenges, including institutional, cultural, financial, technical, and clinical. This article provides an in-depth review of the required elements for establishing such an advanced service, including the associated potential barriers, and shares our experience in establishing the first pelvic exenteration service in Saudi Arabia and the Gulf region.

## 1 Introduction

Pelvic exenteration(PE) has evolved from a radical operation in surgical oncology to a specialized, multidisciplinary service that can offer durable survival and improved quality of life for selected patients with advanced or recurrent pelvic malignancies (1, 2).

During the past decade, the need for structured exenteration programs has grown as advanced pelvic cancers become more common globally. This increased demand highlights ongoing disparities between established high-volume centers and countries with little or no experience in providing coordinated, complex pelvic surgery (3). Many areas of the Middle East still lack the integrated infrastructure needed for advanced pelvic surgery.

Establishing such a service requires far more than the technical capability to perform a complex operation. It requires integrated multidisciplinary pathways, specialized imaging and anesthesia support, in addition to experienced perioperative nursing, stoma and wound care expertise, and administrative support, with a culture that embraces centralization and high-risk oncologic surgery (4).

Centers without previously existing exenteration programs face additional, unique hurdles. These can include limited institutional experience with advanced pelvic surgery, a lack of cross-specialty collaboration, fragmented referral networks, and hesitation among clinicians or patients. Numerous cultural factors may affect the perceptions surrounding stomas, sexuality, and the apprehension regarding postoperative morbidity, which ultimately informs the acceptance and utilization of the service (5). Economic and structural obstacles, encompassing expenditures on technological advancements, intensive care unit capacity, and the recruitment of specialized personnel, further exacerbate the challenges associated with establishing a comprehensive service.

This article is presented as a narrative expert review. A structured, non-systematic literature search was conducted across PubMed/MEDLINE and Scopus to identify relevant English-language publications from 1940 to 2025 on pelvic exenteration, multidisciplinary care models, and program development. It provides an in-depth examination of the challenges and key elements involved in establishing a modern pelvic exenteration service, as well as the authors’ approach on firsthand experience establishing the first dedicated Pelvic Exenteration Service in Saudi Arabia and the Gulf region, this paper details the strategic, technical, cultural, and institutional processes required to deliver high-quality outcomes in a region previously lacking such a service. The insights presented are intended to serve as a practical, adaptable guide for other centers seeking to develop or enhance their advanced pelvic oncology programs.

## 2 Global evolution of exenteration scope

PE was first described as a palliative procedure for advanced cervical cancer confined to the pelvis by Brunschwig in 1948 (6). Later on, in the 1950s–1960s, other surgeons extended the scope of this radical operation to malignancies of other pelvic organs, including the rectum (7). Further evolution occurred with composite bony resections, when Brunschwig and Barber reported the first PE series, including en bloc removal of pelvic bones, in 1969 (8). Sacral resection was later revisited in the 1980s by Wanebo and Marcove (9), whose abdominoprone sacrectomy for posterior pelvic rectal cancer recurrence renewed interest in composite sacral resections and paved the way for more radical procedures in the 1990s–2000s (7). Lateral pelvic sidewall excision also advanced during this period, with Barber and Brunschwig first reporting major iliac vessel resections (10), and subsequent en bloc lateral pelvic wall resections for advanced or recurrent rectal cancer involving neurovascular structures were developed in the early 2000s (7).

## 3 Pelvic exenteration as a service line

PE is more than a single complex operation. It represents an extensive resection that may necessitate the excision of several pelvic organs, bone, vessels, and nerves, and often requires advanced reconstruction (4).

When PE was first introduced, Brunschwig and Bricker operated with a single surgical team, starting with an abdominal approach, followed by a perineal approach (6). However, Schmitz and colleagues subsequently introduced the synchronous two-team abdominoperineal technique in 1959, which has since become standard in modern exenteration units (11).

Multidisciplinary collaboration is key to any exenteration program, which requires structural elements and staffed roles beyond surgeons alone (12).

In the following section, we will discuss the essential elements of a functional exenteration service.

### 3.1 Referral and coordination

A dedicated specialist nurse who receives referrals, streamlines diagnostic workup, schedules all necessary appointments and investigations, and tracks the patient’s journey. This nurse may also coordinate multidisciplinary team (MDT) meetings, where experts discuss patient cases (4).

### 3.2 Specialized imaging and radiology

Experienced pelvic cancer radiologists are essential for high-quality preoperative staging and for evaluating postoperative complications.

A collaborative study highlighted that standardized protocols and specialized radiologists, who provide structured reports for surgeons and preoperative planning, improve patient selection and survival in PE centers (13).

Post-exenteration anatomy can be complex due to surgical changes and complications. Experienced radiologists and protocol-driven approaches are vital to diagnose complications accurately (14, 15).

### 3.3 Formal multidisciplinary team and meeting infrastructure

A dedicated multidisciplinary team meeting where all cases are discussed with the presence of the specialties involved. It is central to decision-making, patient selection, coordination throughout the pathway, and preoperative planning.

In the 1940s (6), Pelvic exenteration began as a single-surgeon procedure. However, over the past few decades, it has evolved into a standardized multidisciplinary cancer operation delivered in specialized units and often discussed in formal MDT meetings (colorectal, urology, gynae, ortho, vascular, plastics, dedicated radiologists, medical and radiation oncologists, histopathologists, clinical nurse specialists) (4).

Published reports from high-volume centers and newer low-volume programs now describe dedicated pelvic exenteration MDTs as the organizational backbone of these services, as they form a critical step in the patient care pathway and are essential to patient selection and resectability decisions (16–18).

Many papers have associated centralization with specialized MDT units with better R0 resection rates and oncological outcomes, noting that even smaller units can still match results when they build structured, dedicated MDTs (4, 19–21). Another study outlined the vital role of MDT-based care for patients undergoing total pelvic exenteration in coordinated planning, holistic needs assessment, and the role of MDT members in reducing short- and long-term morbidity and improving patient satisfaction (4, 18, 22).

A recent study by Pal, A. et al. shed light on PE theatre teams and identified four themes: driving force, technical skills, non-technical skills, and operational aspects. Reflecting how integrated the multidisciplinary skills and processes are, high performance and service delivery underpin regional referral networks, dedicated planning meetings, and specialized perioperative pathways (23).

### 3.4 Experienced surgical specialists

A PE service composed of highly experienced surgical specialists, led by surgeons formally trained in exenteration techniques (4, 18, 23, 24). This leadership is coupled with subspecialists from multiple disciplines, typically including urologic and gynecologic oncology, orthopedic or spinal surgery, vascular surgery, and plastic and reconstructive surgery (4). Although the precise composition of the team varies across institutions, having deep, complementary expertise across these areas is essential to delivering safe multivisceral resections and complex reconstructions in this setting (23, 24).

### 3.5 Medical and radiation oncology integration

Collaboration with medical and radiation oncology is fundamental in any PE unit for multimodal treatment planning, as they play a significant role in determining the patient treatment journey, whether neoadjuvant, adjuvant, or palliative, where appropriate, or by assessing the response and patient selection for PE in those who have failed intensive oncological treatment (4, 16, 18).

These specialists also guide adjuvant systemic therapy and re-irradiation strategies after surgery, support clinical trial enrolment, and contribute to long-term survivorship planning, reinforcing that exenteration services function best as integrated oncologic programs rather than stand-alone surgical platforms.

### 3.6 Perioperative support

Peri-operative support in PE plays a pivotal role in pre-operative preparation and post-operative management to achieve better clinical outcomes (4, 18, 25).

Starting with the anesthetist assessment and pre-operative evaluation of patients before PE surgery, to determine the patient’s fitness and functional reserve (4, 25). In addition to identifying potential risk factors that may increase perioperative risk, such as cardiac or respiratory conditions, and working to optimize comorbid conditions when possible (4, 25).

Moreover, the need for Seasoned anesthesiologists familiar with lengthy, high-risk pelvic procedures associated with high blood loss is very crucial to such a complex operation. With appropriate integration of hemodynamic and respiratory intraoperative monitoring modalities, a comprehensive evaluation of the patient’s hemodynamic status can be achieved to facilitate intraoperative decision-making (25). A dedicated an aesthetic team for exenterative procedures can centralize expertise and clinical experience and ensure a smooth workflow (25).

### 3.7 Adequate ICU or high-dependency capacity

Immediate access to the intensive care units is a core requirement for a PE program, highlighting the operation’s long duration, major fluid shifts, and high risk of early complications such as sepsis, bleeding, and respiratory failure (26).

Several clinical reviews recommend routine postoperative transfer to an ICU or high-dependency unit for close hemodynamic monitoring, ventilatory support, and aggressive management of pain, nutrition, and anticoagulation in the first days after surgery (4, 25, 26). This standard practice of keeping exenteration patients in ICU or critical-care environments for several days underscores that robust critical-care service and capacity are integral to safe service delivery and management of the substantial morbidity associated with this procedure (4, 25, 26).

### 3.8 Pathology services

A high-quality PE unit needs a dedicated pathology service experienced in handling complex operative specimens and their anatomical orientation. As per many published studies, long-term outcomes after pelvic exenteration are driven by meticulous assessment of resection margins, lymph nodes, tumor extent, grade, and stage, which underlines how closely surgical quality and service benchmarking depend on robust, standardized pathological input (27, 28). Achieving an R0 resection is considered the single most crucial pathological determinant of disease-free and overall survival after exenteration (28), making accurate margin evaluation (including circumferential and lateral pelvic sidewall margins) crucial for both prognostication and adjuvant treatment planning. Many high-volume centers are advocating for standardizing the synoptic reporting system for pelvic exenteration patients, as it will lead to more consistent and complete pathology reporting, resulting in improved patient management (27).

### 3.9 Rehabilitation and allied health

A structured rehabilitation program and physiotherapy are the cornerstones of postoperative care after PE. The aim is to help patients improve their mobility and functional independence after such a morbid operation. Early focused physical therapy can help reduce postoperative complications, improve lower-limb strength, and enhance daily activities following PE (29). This will be part of enhanced recovery and critical-care pathways for PE, in addition to respiratory exercises and progressive gait training, which are associated with better functional capacity, shorter ICU or hospital stay, and improved health-related quality of life in the months after surgery (29). This can be achieved through inpatient physiotherapy and step-down rehabilitation services to support long-term recovery of walking ability and everyday function.

### 3.10 Psychiatric and psycho-oncology support

The radicality of this surgery can profoundly have an impact on body image, sexuality, social roles, and overall psychological well-being; therefore, psychiatric and psycho-oncology support is a critical component of pelvic exenteration programs. Both preoperatively (to prepare patients for the life-changing impact of stomas, reconstructive procedures, and functional loss) and long-term (to support coping, intimacy, and adaptation to a “new normal”) (30, 31).

### 3.11 Dietitians for nutritional optimization before and after surgery

The patients’ cohort undergoing PE surgery can be in poor nutritional status, which is strongly associated with higher complication rates, longer hospital stay, and worse quality of life (32–34). Dedicated nutrition screening and preoperative optimization by specialist dietitians, in addition to post-operative enteral or parenteral support, are core components of prehabilitation pathways for exenteration candidates (32, 34).

### 3.12 Specialized nursing and wound care

Expert stoma and wound care nurses are fundamental in the PE unit to manage ostomies, wound dehiscence, and complex perineal pelvic wounds. They can also provide preoperative stoma education and long-term follow-up to help patients manage new ostomies and complex pelvic wounds, which is now standard practice in major exenteration centers (22).

In addition, experienced oncology nursing staff across operating theatres and surgical wards are essential to deliver advanced patient care in this very high-acuity patient group.

### 3.13 Centralization of care and regional specialization

Recent publications from high-volume centers highlighted the shift toward performing PE in centralized care in specialized units, which led to improved multidisciplinary input, integration of prehabilitation and subspecialty surgery, and enabled research and QoL assessment (19, 35). Centralization of this service to high-volume specialized centers is linked to better perioperative and oncological outcomes, including lower mortality (20, 36, 37).

Selective PE performed at lower-volume tertiary centers, when supported by robust multidisciplinary infrastructure, can deliver results similar to those reported by large centers, reinforcing a strategy of regional specialization with a structured referral pathway (20, 37).

## 4 Why the institution matters

A successful exenteration service typically sits within a tertiary referral center where other complex surgeries are already part of routine practice. This existing complexity makes it easier to assemble and coordinate the multidisciplinary team, because many specialists already have experience managing complex cases.

However, pelvic exenteration still differs from other complex surgeries in two keyways:

- Relative unfamiliarity: Even in high-level centers, many clinicians and staff may lack routine exposure to exenteration, requiring deliberate education, protocols, and mentorship.
- High institutional commitment: The program demands sustained administrative support, investment in personnel and infrastructure, and a culture that embraces centralized, high-risk oncologic surgery rather than *ad hoc* or fragmented efforts.

## 5 Barriers and challenges to establishing a new exenteration program

### 5.1 Financial and institutional barriers

Establishing a new pelvic exenteration center is frequently constrained by substantial financial and institutional barriers, as the procedure is a low-volume, high-cost intervention that demands prolonged operating times, routine ICU admission, and extended hospital stays, all of which drive up total inpatient and staffing costs. Hospital administrators may have limited awareness of contemporary outcomes and cost-effectiveness data. They therefore may perceive exenteration as excessively risky or not financially viable, creating resistance to funding new programs despite evidence that centralized, well-resourced units can achieve good long-term outcomes and more efficient resource use over time (38–42).

### 5.2 High upfront and ongoing cost (infrastructure, ICU, equipment, staffing)

An extensive Australian cost analysis study of pelvic exenterations reports a median in-hospital admission cost of approximately AUD $108,000, with staffing and operating room costs the highest (39). According to this analysis, they conclude that the cost “reflects the complexity of the procedure and the multidisciplinary requirement” (39). In another global PelvEx Collaborative study, the study estimates the global in-hospital perioperative cost at around USD $55,000 per exenteration, based on imaging, a long operation, ICU stay, and 14 days on the ward, and highlights that reconstruction and rehabilitation are “both time and resource-consuming” (40). Moreover, the study stresses that indirect costs, such as physical therapy, complications, readmissions, and rehabilitation, further increase expenditure beyond the direct theatre and bed-day costs (40).

Such studies only support the fact that costly infrastructure, ICU capacity, long operative times, highly specialized multidisciplinary surgeons, nursing, and allied health staff are significant cost barriers for most hospitals.

### 5.3 High-cost, low-volume nature, and need for institutional buy-in

For any new PE service, the nature of this service, with a “low-volume, high-cost surgical program,” presents unique health management challenges, and any successful establishment will require organization-wide support and a proactive, collaborative relationship with hospital administration and management (38).

### 5.4 Technical and clinical barriers

Establishing a PE service can be hindered by some technical and clinical barriers that extend beyond infrastructure. A significant limitation is the shortage of surgeons with formal experience in exenteration, as training opportunities are primarily confined to a few high-volume tertiary centers. Added to this expertise gap is the historically, and partially still, present fragmentation of clinical practices among the colorectal, gynecologic oncology, and urology disciplines, which can impede coordinated team approaches essential for complex multivisceral resections. Furthermore, many hospitals lack access to specialized radiologists capable of detailed pelvic mapping, including owning high-resolution MRI and PET-CT, which are critical for assessing resectability and surgical planning (22, 43). Finally, the postoperative phase demands complex wound management expertise as well as structured rehabilitation for physical and psychological recovery resources frequently unavailable outside established exenteration centers, limiting safe program development and adequate patient outcomes (22, 43).

### 5.5 Cultural and social barriers

Cultural and social factors can significantly shape perceptions of PE. Many patients are hesitant to consider this extensive surgery because of fears about living with one or more stomas, significant changes in body image, and the impact on sexuality (5, 30, 31).

Misconceptions among clinicians, with some still viewing exenteration as essentially a palliative with uniformly poor quality of life, can lead to under-referral or delayed referral to specialist centers, despite evidence of long-term survival in selected patients (44). Reports from newer programs suggest that limited awareness of regional exenteration services among peripheral hospitals further contributes to referral delays, reinforcing the importance of proactive outreach and culturally sensitive counselling that address individual beliefs, family dynamics, and expectations (44).

## 6 Building the first exenteration service in Saudi Arabia and the Gulf region: our experience

King Faisal Specialist Hospital & Research Centre (KFSH&RC) is among the leading tertiary and quaternary referral hospitals in the Middle East. It is a highly specialized institution with advanced technology that primarily manages complex tertiary and quaternary cases across a wide range of specialties, including comprehensive cancer care.

The establishment of a pelvic exenteration service within an institution already managing highly complex oncologic cases was relatively seamless. Soft-tissue pelvic exenterations, including total pelvic exenteration or two-compartment pelvic exenterations in female patients, have been performed for over 15 years. Over the past five years, the service has expanded to more extended pelvic exenteration procedures requiring bony resections, such as sacrectomies and pubic bone resections. These cases remain relatively infrequent and are performed within a structured multidisciplinary framework. Detailed case numbers and oncologic outcomes are being reported separately in a dedicated outcomes analysis. KFSH&RC is the first center in the Middle East to join the PelvEx Collaborative, contributing regional data and participating in an international collaborative to advance the practice of pelvic exenteration.

All necessary multidisciplinary specialties required for these complex procedures were already available, including an experienced anesthetic team accustomed to prolonged operations with significant blood loss, all other surgical specialties, including ortho-oncology and spine surgery, urology, gynecologic oncology, vascular, plastic, specialized intensive care unit staff, expert radiologists, and highly trained nursing teams. The service was developed in accordance with well-established international standards for PE, emphasizing rigorous patient selection and incorporating structured preoperative optimization, standardized operative planning, and comprehensive postoperative management (4).

As a well-established tertiary institution, KFSH&RC did not face significant financial or technical barriers in implementing this service. However, cultural and organizational challenges were encountered.

These included variability in referral patterns and misperceptions among clinicians regarding acceptable morbidity in pursuit of oncologic clearance and the feasibility of the pelvic exenteration procedure as a curative procedure. In addition, patient- and family-centered cultural expectations necessitated enhanced counselling and shared decision-making, particularly when discussing the magnitude of surgery, the likelihood of permanent stomas, and potential functional outcomes.

Other challenges relate to patient-level factors, as a large proportion of our cohort has multiple comorbidities that can make them higher risk for this surgery, which further narrows selection criteria, and this is compounded by the high prevalence of obesity and sedentary lifestyle in the Saudi population, where 38% of individuals are overweight, 20% are obese and 80% are physically inactive (45).

However, achieving true centralization of care at the national and regional levels requires strengthening referral pathways and increasing clinician awareness. Surgeons and medical oncologists should be encouraged to seek early second opinions from specialized centers rather than independently determining unresectability or categorizing patients as palliative. Reducing specialty silos and fostering collaborative decision-making are essential steps toward ensuring that patients with potentially resectable disease receive appropriate expert evaluation.

Addressing these barriers required sustained institutional engagement, education of referring teams, and the development of a dedicated pelvic exenteration multidisciplinary team (MDT) to ensure consistent messaging, transparent risk–benefit discussions, and alignment of surgical intent with patient values.

Given that Colorectal cancer is the second most common cancer in Saudi Arabia (45), the regional demand for a specialized center capable of delivering advanced pelvic exenteration within a structured MDT framework positioned KFSH&RC as the preferred referral hub, supported by its existing expertise and dedicated resources.

## 7 Conclusion

Developing a PE program might transform cancer care within any organization, especially in regions where such services have not previously existed. From our Experience as a newly established service in Saudi Arabia and the Gulf region, we demonstrate that success relies on several foundational principles that can guide institutions aspiring to develop similar programs. Strong leadership anchored by a committed surgeon who can champion the service is essential. Centralization of care is considered a crucial factor, ensuring that patients with advanced pelvic cancers receive multidisciplinary evaluation, with direct access to highly specialized expertise.

Equally important is to avoid common pitfalls, such as undertaking exenterations without structured pathways or adequate multidisciplinary input, which protects both patient outcomes and long-term program viability. Early, small achievements, whether structured referral pathways, successful initial cases, or improved perioperative processes, help demonstrate value and gain administrative buy-in.

These insights naturally flow into a practical blueprint for program development. Institutions should begin by evaluating population needs and case load, followed by securing leadership endorsement and institutional commitment. Building the core surgical team, standardizing MDT and radiologic pathways, and initiating practice with carefully selected early cases provide a stable foundation. This stepwise model is adaptable across different health organizations, including those with limited resources.

Finally, exenteration programs represent more than a surgical service; they reflect a comprehensive, advanced pelvic cancer care, which can significantly improve survival and quality of life for these patients. Our experience in Saudi Arabia and the Gulf region shows that even in settings new to exenteration, a structured, collaborative, and data-driven approach can yield a program that is both clinically impactful and globally relevant.

## References

[1] Kuhrt MP Chokshi RJ Arrese D Martin EW Jr . Retrospective review of pelvic Malignancies undergoing total pelvic exenteration. World J Surg Oncol. (2012) 10:110. doi: 10.1186/1477-7819-10-110, PMID: 22703863 PMC3465228

[2] PelvEx Collaborative . Surgical and survival outcomes following pelvic exenteration for locally advanced primary rectal cancer: results from an international collaboration. Ann Surg. (2019) 269(2):315–21. doi: 10.1097/SLA.0000000000002528, PMID: 28938268

[3] Brown KGM Solomon MJ Koh CE Sutton PA Aguiar S Jr. Bezerra TS . Defining benchmarks for pelvic exenteration surgery: A multicentre analysis of patients with locally advanced and recurrent rectal cancers. Ann Surg. (2025) 282:1118–26. doi: 10.1097/SLA.0000000000006348, PMID: 38747145

[4] PelvEx Collaborative . Minimum standards of pelvic exenterative practice: PelvEx Collaborative guideline. Br J Surg. (2022) 109(12):1251–63. doi: 10.1093/bjs/znac317, PMID: 36170347

[5] Turns D . Psychosocial issues: pelvic exenterative surgery. J Surg Oncol. (2001) 76:224–36. doi: 10.1002/jso.1036, PMID: 11276026

[6] Brunschwig A . Complete excision of pelvic viscera for advanced carcinoma; a one-stage abdominoperineal operation with end colostomy and bilateral ureteral implantation into the colon above the colostomy. Cancer. (1948) 1:177–83. doi: 10.1002/1097-0142(194807)1:2<177::AID-CNCR2820010203>3.0.CO;2-A 18875031

[7] Brown KGM Solomon MJ Koh CE . Pelvic exenteration surgery: the evolution of radical surgical techniques for advanced and recurrent pelvic Malignancy. Dis Colon Rectum. (2017) 60:745–54. doi: 10.1097/DCR.0000000000000839, PMID: 28594725

[8] Brunschwig A Barber HR . Pelvic exenteration combined with resection of segments of bony pelvis. Surgery. (1969) 65:417–20. 5765364

[9] Wanebo HJ Marcove RC . Abdominal sacral resection of locally recurrent rectal cancer. Ann Surg. (1981) 194:458–71. doi: 10.1097/00000658-198110000-00009, PMID: 7283507 PMC1345323

[10] Barber HR Brunschwig A . Excision of major blood vessels at the periphery of the pelvis in patients receiving pelvic exenteration: common and/or iliac arteries and veins 1947 to 1964. Surgery. (1967) 62:426–30. 6038178

[11] Schmitz HE Schmitz RL Smith CJ Molitor JJ . The technique of synchronous (two team) abdominoperineal pelvic exenteration. Surg Gynecol Obstet. (1959) 108:351–6. doi: 10.1097/00006254-195908000-00048, PMID: 13635246

[12] Brown KGM Morkaya J Solomon MJ Ng KS White K Sutton P . Priority outcomes of pelvic exenteration for rectal cancer: a patient, carer, and clinician consensus. Br J Surg. (2024) 111. doi: 10.1093/bjs/znae298, PMID: 39656602 PMC11631376

[13] Nougaret S Lambregts DMJ Beets GL Beets-Tan RGH Blomqvist L Burling D . Imaging in pelvic exenteration-a multidisciplinary practice guide from the ESGAR-SAR-ESUR-PelvEx collaborative group. Eur Radiol. (2025) 35:2681–91. doi: 10.1007/s00330-024-10940-z, PMID: 39181949 PMC12021987

[14] Guha A Gandhi S Mynalli S Baheti A Haria P Choudhari A . A radiologist’s guide to the galaxy of complications post total pelvic exenteration for rectal cancers. Clin Radiol. (2025) 80:106719. doi: 10.1016/j.crad.2024.10.002, PMID: 39579393

[15] Sbarra M Miccò M Corvino M Persiani S Gui B Di Paola V . CT findings after pelvic exenteration: review of normal appearances and most common complications. Radiol Med. (2019) 124:693–703. doi: 10.1007/s11547-019-01009-9, PMID: 30806919

[16] O’Shannassy SJ Brown KGM Steffens D Solomon MJ . Referral patterns and outcomes of a highly specialised pelvic exenteration multidisciplinary team meeting: A retrospective cohort study. Eur J Surg Oncol. (2020) 46:1138–43. doi: 10.1016/j.ejso.2020.02.031, PMID: 32122755

[17] Fitzsimmons T Thomas M Tonkin D Murphy E Hollington P Solomon M . Establishing a state-wide pelvic exenteration multidisciplinary team meeting in South Australia. ANZ J Surg. (2023) 93:1227–31. doi: 10.1111/ans.18220, PMID: 36567641

[18] Kontovounisios C Tekkis P . Locally advanced disease and pelvic exenterations. Clin Colon Rectal Surg. (2017) 30:404–14. doi: 10.1055/s-0037-1606118, PMID: 29200980 PMC5711537

[19] Lampe B Luengas-Würzinger V Weitz J Roth S Rawert F Schuler E . Opportunities and limitations of pelvic exenteration surgery. Cancers (Basel). (2021) 13(24):6162. doi: 10.3390/cancers13246162, PMID: 34944783 PMC8699210

[20] Mackowski A Limmer A McCarthy A Gilmore A . Pelvic exenteration surgery outcomes following establishment of a complex pelvic surgery multidisciplinary tertiary unit. Gen Surg Clin Med. (2024) 2. doi: 10.21203/rs.3.rs-3455761/v1, PMID: 41716896

[21] Traeger L Bedrikovetski S Oehler MK Cho J Wagstaff M Harbison J . Short-term outcomes following development of a dedicated pelvic exenteration service in a tertiary centre. ANZ J Surg. (2022) 92:2620–7. doi: 10.1111/ans.17921, PMID: 35866328 PMC9795898

[22] Carvalho F Qiu S Panagi V Hardy K Tutcher H MaChado M . Total Pelvic Exenteration surgery - Considerations for healthcare professionals. Eur J Surg Oncol. (2023) 49:225–36. doi: 10.1016/j.ejso.2022.08.011, PMID: 36030135

[23] Pal A Chittleborough T McCombie A Glyn T Frizelle FA . Human factors in pelvic exenteration: themes in high-performing teams. Colorectal Dis. (2024) 26:95–101. doi: 10.1111/codi.16825, PMID: 38057630

[24] Collaborative GPE . Pelvic Exenteration for locally advanced rectal cancer (LARC) and locally recurrent rectal cancer (LRRC) in Greece: A national snapshot survey of current practice. Hell J Surg. (2024) 94:115–41. doi: 10.59869/24037, PMID: 41233906

[25] PelvEx Collaborative . Perioperative management and anaesthetic considerations in pelvic exenterations using Delphi methodology: results from the PelvEx Collaborative. BJS Open. (2021) 5(1):zraa055. doi: 10.1093/bjsopen/zraa055, PMID: 33609393 PMC7893479

[26] Ng KS Lee PJM . Pelvic exenteration: Pre-, intra-, and post-operative considerations. Surg Oncol. (2021) 37:101546. doi: 10.1016/j.suronc.2021.101546, PMID: 33799076

[27] Däster S Shin JS Loizides S Steffens D Koh CE Solomon MJ . Pathology reporting of pelvic exenteration specimens for locally recurrent rectal cancer. Eur J Surg Oncol. (2021) 47:2100–7. doi: 10.1016/j.ejso.2021.03.258, PMID: 33895021

[28] Gould LE Pring ET Drami I Moorghen M Naghibi M Jenkins JT . A systematic review of the pathological determinants of outcome following resection by pelvic exenteration of locally advanced and locally recurrent rectal cancer. Int J Surg. (2022) 104:106738. doi: 10.1016/j.ijsu.2022.106738, PMID: 35781038

[29] Steffens D Young JM Solomon M Beckenkamp PR Koh C Vuong K . Preliminary evidence for physical activity following pelvic exenteration: a pilot longitudinal cohort study. BMC Cancer. (2019) 19:661. doi: 10.1186/s12885-019-5860-5, PMID: 31272406 PMC6610976

[30] Andersen BL Hacker NF . Psychosexual adjustment following pelvic exenteration. Obstet Gynecol. (1983) 61:331–8. PMC2722222 6823375

[31] Harji DP Griffiths B Velikova G Sagar PM Brown J . Systematic review of health-related quality of life in patients undergoing pelvic exenteration. Eur J Surg Oncol. (2016) 42:1132–45. doi: 10.1016/j.ejso.2016.01.007, PMID: 26968226

[32] Hogan S Steffens D Vuong K Rangan A Solomon M Carey S . Preoperative nutritional status impacts clinical outcome and hospital length of stay in pelvic exenteration patients - a retrospective study. Nutr Health. (2022) 28:41–8. doi: 10.1177/02601060211009067, PMID: 33858255

[33] van Rees JM Visser E van Vugt JLA Rothbarth J Verhoef C van Verschuer VMT . Impact of nutritional status and body composition on postoperative outcomes after pelvic exenteration for locally advanced and locally recurrent rectal cancer. BJS Open. (2021) 5. doi: 10.1093/bjsopen/zrab096, PMID: 34672343 PMC8529522

[34] Watt A Kaushik V Harris C Yeung CH Lam YN Osland E . Nutrition-related predictors of complications and length of hospital stay following total pelvic exenteration surgery. Clin Nutr ESPEN. (2024) 62:88–94. doi: 10.1016/j.clnesp.2024.05.005, PMID: 38901953

[35] PelvEx Collaborative . Contemporary management of locally advanced and recurrent rectal cancer: views from the pelvEx collaborative. Cancers (Basel). (2022) 14(5):1161. doi: 10.3390/cancers14051161, PMID: 35267469 PMC8909015

[36] Venchiarutti RL Solomon MJ Koh CE Young JM Steffens D . Pushing the boundaries of pelvic exenteration by maintaining survival at the cost of morbidity. Br J Surg. (2019) 106:1393–403. doi: 10.1002/bjs.11203, PMID: 31282571

[37] Humphries EL Kroon HM Dudi-Venkata NN Thomas ML Moore JW Sammour T . Short- and long-term outcomes of selective pelvic exenteration surgery in a low-volume specialized tertiary setting. ANZ J Surg. (2019) 89:E226–e30. doi: 10.1111/ans.15212, PMID: 31067602

[38] Brown KGM McBride KE Anderson T Solomon MJ . Delivering complex surgical services: lessons learned from the evolution of a specialised pelvic exenteration centre. Aust Health Rev. (2023) 47:735–40. doi: 10.1071/AH23186, PMID: 38029447

[39] Risbey CWG Brown KGM Solomon M McBride K Steffens D . Cost analysis of pelvic exenteration surgery for advanced pelvic Malignancy. Ann Surg Oncol. (2024) 31:9079–87. doi: 10.1245/s10434-024-16227-3, PMID: 39284989 PMC11549131

[40] PelvEx Collaborative . The global cost of pelvic exenteration: in-hospital perioperative costs. Br J Surg. (2020) 107(11):e470–e1. doi: 10.1002/bjs.11924, PMID: 33460051

[41] Guo Y Chang E Bozkurt M Park M Liu D Fu JB . Factors affecting hospital length of stay following pelvic exenteration surgery. J Surg Oncol. (2018) 117:529–34. doi: 10.1002/jso.24878, PMID: 29044540 PMC5854513

[42] Koh CE Badgery-Parker T Salkeld G Young JM Heriot AG Solomon MJ . Cost-effectiveness of pelvic exenteration for locally advanced Malignancy. Br J Surg. (2016) 103:1548–56. doi: 10.1002/bjs.10259, PMID: 27559684

[43] PelvEx Collaborative . Changing outcomes following pelvic exenteration for locally advanced and recurrent rectal cancer. BJS Open. (2019) 3:516–20. doi: 10.1002/bjs5.50153, PMID: 31388644 PMC6677093

[44] Dessai SB Balasubramanian S Patil VM Chakraborty S Bhattacharjee A Vikram S . Pelvic exenteration: experience from a rural cancer center in developing world. Int J Surg Oncol. (2015) 2015:729658. doi: 10.1155/2015/729658, PMID: 25741445 PMC4337038

[45] Alessa AM Khan AS . Epidemiology of colorectal cancer in Saudi Arabia: A review. Cureus. (2024) 16:e64564. doi: 10.7759/cureus.64564, PMID: 39144848 PMC11323712

